# Petrus Leo: a contribution to the art of uroscopy

**DOI:** 10.1007/s40620-021-01088-w

**Published:** 2021-06-17

**Authors:** Massimo Torreggiani, Marco Colucci, Carlo Enrico Confalonieri, Ciro Esposito

**Affiliations:** 1 Nephrology and Dialysis, Centre Hospitalier Le Mans, 194 Avenue Rubillard, 72037 Le Mans, France; 2 Unit of Nephrology and Dialysis, ICS Maugeri s.p.a. SB, Pavia, Italy; 3 University of Pavia, Pavia, Italy

**Keywords:** Petrus Leo, De urinis, Murder, Uroscopy, Lorenzo The Magnificent, Renaissance, Italy, Middle age

## Introduction

**Fig. 1:**
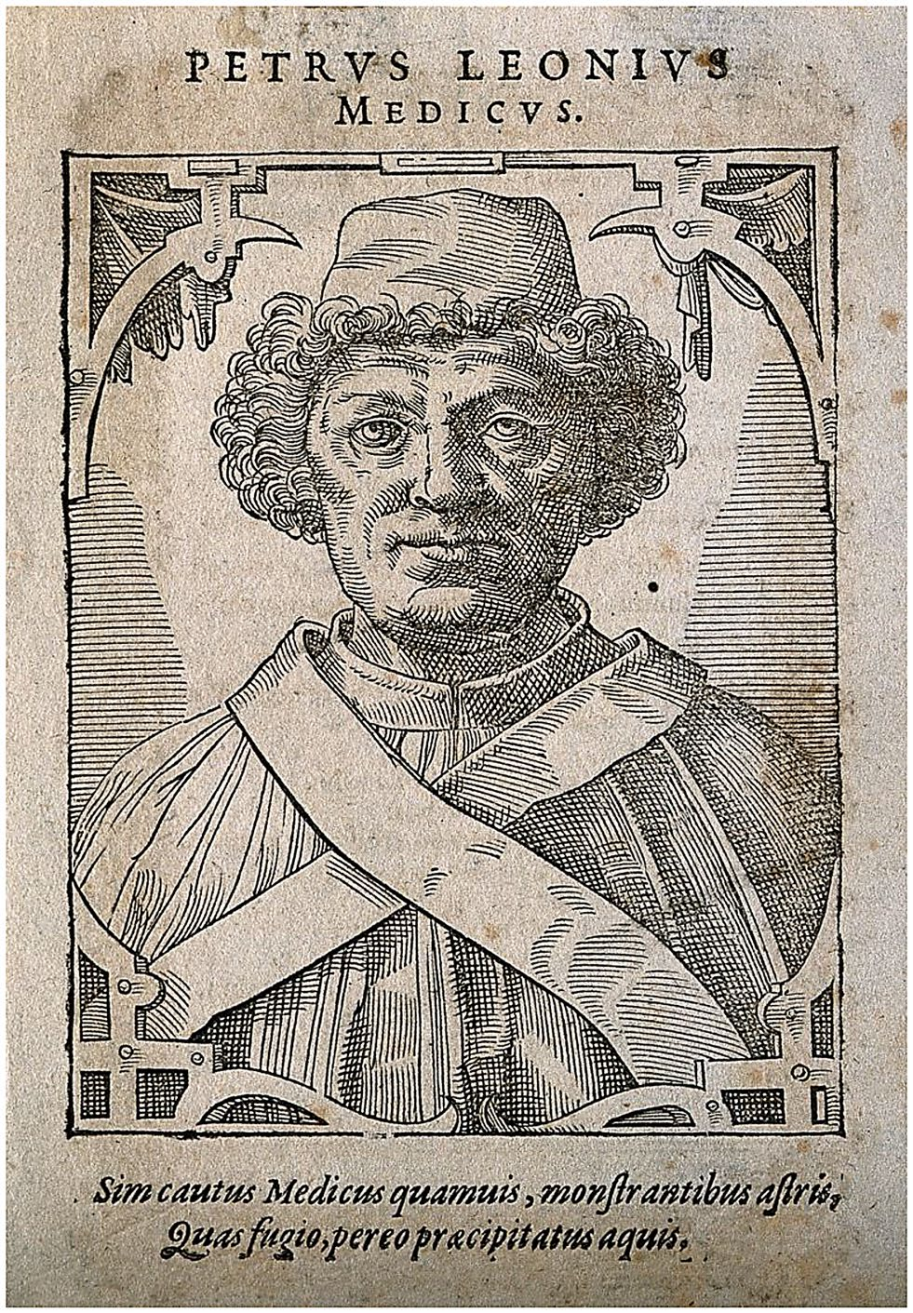
Petrus Leo (from Pietro Leoni. Woodcut by T. Stimmer, 1589. Wellcome Collection. Public Domain Mark). Petrus Leo (Spoleto, 1445 – Florence, 9 April 1492) studied medicine in Rome. He was professor of medicine for the universities of Pisa and Padua and Lorenzo de’ Medici personal doctor. He was part of the Neoplatonic circle, alongside Marsilius Ficinus and Giovanni Pico della Mirandola

Petrus Leo (Fig. 1), an eminent physician of the Renaissance, was Lorenzo de’ Medici’s (“the Magnificent”) personal doctor (see Supplementary material for a biographical sketch). On January 18, 1478 Petrus Leo finished to write his treatise about uroscopy, entitled *De urinis* [1, 2]. This body of work took form during his teaching in Pisa but was published only after his death by a Venetian publisher, Mutius Avenantius, as an appendix to a larger treatise by Egide de Corbeil: “*De urinis et pulsibus*” [3]. Here, we discuss, for the first time in English, Petrus Leo’s work on uroscopy.

## *De urinis* [3]

In the *De urinis* introduction, Petrus acknowledges a number of authoritative treaties about urines and, with a rhetorical expedient, agrees with those who would call him fool to write the umpteenth essay on this topic. However, he notices that Galen and Hippocrates, the most illuminated physicians, never compiled specific works on this subject and thus this lack was to be filled. In this *incipit* there is a strong will to recover the Greek classics and Galen. Indeed, the explicit aim of Leo’s work, as stated in the *prohemium*, was “to give a general example which clearly illustrates the form of Galen’s scientific method. With these premises, it will be possible to get a clear, methodical and tidy insight in detail into the urological matter” [4]. He invites the reader to focus not only on the urinary matter but mostly on the methodology, applicable to other medical topics and providing the instruments to comprehend Galen work and analyze any other concept of medicine.

Following, Petrus divides his treatise into nine chapters, organized in three sections: urine definition, significance of the different urine appearances, association of different diseases to their urine counterpart.

Leo defines the term urine stating that it is called so “either because it is the only product of kidneys or from the Latin verb *uro*, that means to burn, or from the Greek word *urat*, that in Latin means description”. For Leo, urine is nothing more than what exit from a curable body through the urethra or another similar organ, from bladder and kidneys and is produced by the liver by means of digestion. Right after that, a criticism to previous Authors: urine is not a blood leachate as per Theophilus’ definition, nor serum as in Egide’s vision. However, Petrus does not completely reject the previous theories and tries to reach to a synthesis by accepting that an “essential” urine is produced by the liver and that a second or “accident” urine forms into the kidneys and gets mixed with the main one. This postulate proceeds from two observations: first, urine becomes apparent only after its passage through the kidneys and, second, when kidneys are ill but the liver still functions, urine is produced in normal quantity and its appearance does not change. The contrary is not valid since when the liver is sick, it produces an undigested urine. Among body secretions, urine is the preeminent one, and has a universal value to diagnose diseases and formulate prognosis. Thus, urine can be compared to the examination of pulse, first medical evaluation when visiting a patient. In Leo’s view, urine is a mandatory evaluation because, resulting from an overabundance of blood, it comes in contact with all the organs as they are perfused by blood and its characteristics reflect the perfused body system, whose health or disease state is able to modify urine composition and aspect.

In the second chapter, Petrus gives advices to standardize the examination. For instance, the patient must be in a quiet state of mind as any emotion could potentially alter the properties of urine and invalidate or mislead the examination. Urine must not be collected at night. Moreover, the collection vial must have a specific form: it should be a clear, crystalline glass, quite large, with a round bottom (the so called “*matula*” of traditional literature [5]). Urine must be inspected in the place of production and in the vial of collection, it must not be moved to other places nor shacked or transferred to different containers. The evaluation must be performed in a sunny environment not before 30 min and within six hours from collection.

According to Petrus, there are eight different primary features of the urine (*genera differentiarum*): substance, quantity, quality, transparency, odor, taste, way of voiding, color and content. Actually, Leo considers that taste is not really relevant but recognizes the importance of including it in an exhaustive dissertation for scientific purposes. Each *genus* can be further subdivided into several traits (*differentiae specificae*). For example, for substance, there are three possibilities: fine, thick or moderate. Quantity, instead, comprises quantitative and qualitative measures: volume of single voiding (abundant, poor, moderate) and voiding frequency (high or low) [6].

The most striking innovation of Leo’s work, is the expansion of the classical uroscopy wheel: he recognizes 42 possible urine colors, 22 more than those suggested by the traditional uroscopy, even if he acknowledges that this has limited clinical application. The last feature, content, includes all the contaminants that can be found in urines and identifies the most important one to come to a diagnosis: the sediment (*hypostasis*).

**Fig. 2:**
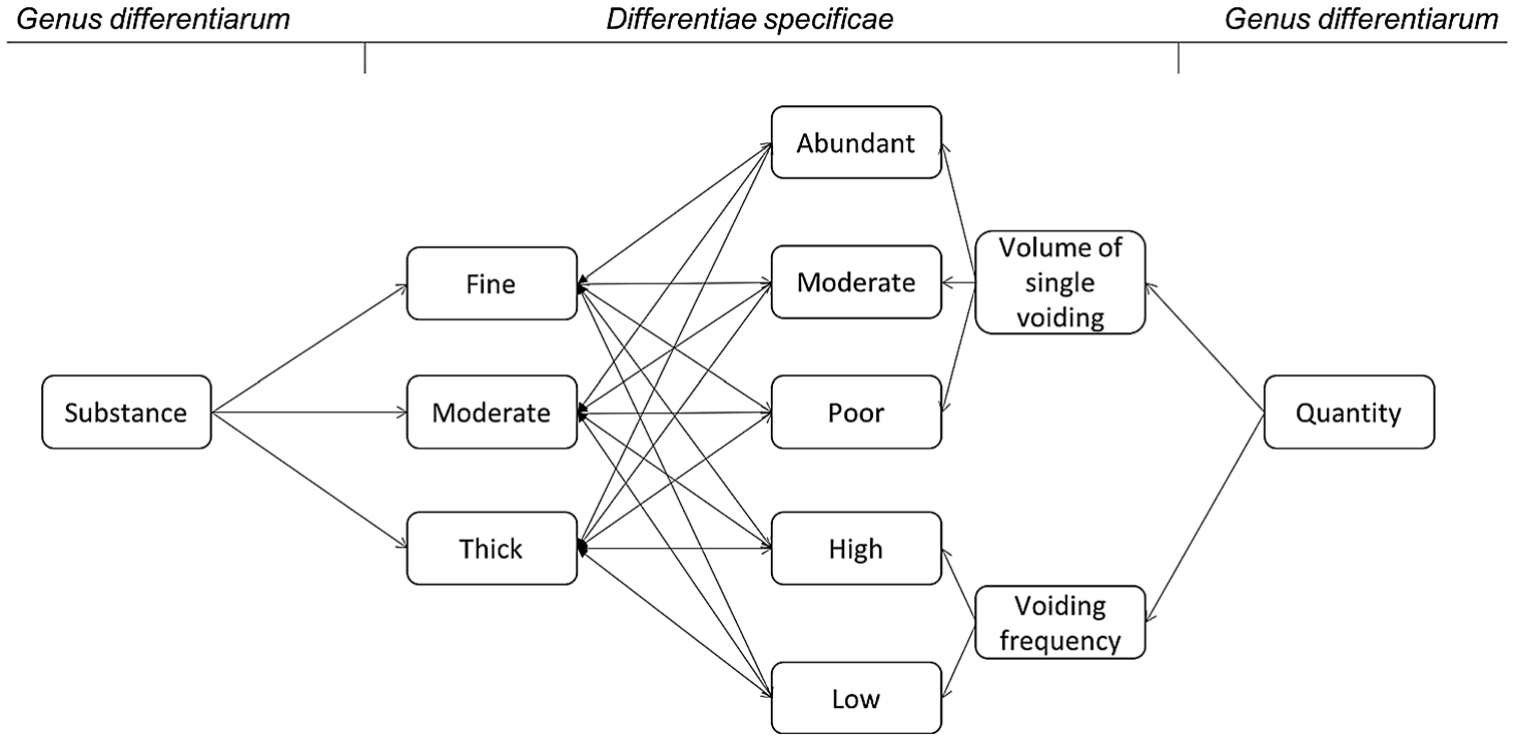
Example of the *ars combinatoria* applied to uroscopy in Petrus Leo’s De urinis

Besides the number of characteristics attributed to urine, it is important to note the innovative methodology. Applying the Lullian theories (see Supplementary material), each feature can be associated with the others in an almost infinite number of combinations. Starting from combining features inside the same genre, such as stones and foam (content), it is possible to further combine different subcategories of other genres. The mathematical system can be used in a binary, tertiary, quaternary (etc.) way: for example the properties of one *genera differentiarum* can be combined with the *differentiae specificae* of all the other *genera* creating couples, trilogies etc. in a finite but vast number of possibilities (Fig. 2). This approach should grant completeness.

Such a systematic description fuses mathematical and philosophical theories and it is the strength and limitation of Leo’s work. The Author is well aware of the complexity limiting daily application and thus suggests identifying the main characteristics as first step, since an accurate diagnosis may be based just on three to four of them.

The second part of the treatise deals with the causes that modify urines: every perturbation of the balance between the humoral system, the four different primary humors (*complexion*) and the digestive process can influence urine excretion and properties.

Finally, in the third part, Petrus enumerates the most common diseases and their effect on urines. Moreover, he drafts the correspondence between the different sections of the urinary vial and the different parts of the body affected by a disease and the opportunity to understand whether the disease is at its beginning or toward its end.

The final chapter is dedicated to the prognosis that can be inferred from urine evaluation. The ability to formulate a prognosis was what gave a doctor his prestige; for this reason Leo invites the reader to treasure this final section.

## Commentary

Petrus Leo was a prominent physician of the Renaissance. His medical knowledge reflected his time, characterized by the rediscovery of Greek classics in opposition to Arabian medicine. His interests spanned from medicine to astrology; he belonged to the Neoplatonics that gathered around Marsilius Ficinus and Lorenzo de’ Medici.

Leo’s contribution to its contemporary medicine was minor with respect to other figures like Egide de Corbeil. Indeed, the methodological approach proposed by Petrus fits better with a theoretical discussion rather than to practical evaluation. Nevertheless, Petrus Leo had the merit to try to uniform the art of uroscopy, i.e. the only examination of body fluids available in that time, and to clearly identify a logic pathway from diagnosis to prognosis.

## Supplementary Information

Below is the link to the electronic supplementary material.

Supplementary material 1 (DOCX 47.9 kb)

## References

[1] Rotzoll M (2000). Pierleone da Spoleto: vita e opere di un medico del Rinascimento. Accademia La Colombaria. Serie studi.

[2] Cod Vat Ross 672 (X 52), cart. misc., c 116

[3] de Corbeil G, Leo P (1514) Egidius de urinis et pulsibus, Petrus Leo de urinis. Avenantius, Mutius, Venetia

[4] Leo P (1514) De urinis. Mutius Avenantius, Venezia

[5] Eknoyan G (2007). Looking at the urine: the renaissance of an unbroken tradition. Am J Kidney Dis.

[6] Rotzoll M (2000). Pierleone da Spoleto: vita e opere di un medico del Rinascimento. Accademia La Colombaria. Serie studi.

