# Supplementary material for: Petrus Leo: a contribution to the art of uroscopy
Source: J Nephrol. 2021 Jun 17;35(2):693–5. doi: 10.1007/s40620-021-01088-w (PMC8927029; doi:10.1007/s40620-021-01088-w)
Supplement: Supplementary file 1 — Supplementary material 1 (DOCX 47.9 kb) [file 40620_2021_1088_MOESM1_ESM.docx]

**Petrus Leo: a contribution to the art of uroscopy.**

Massimo Torreggiani^1,*,‡^ and Marco Colucci^2,‡^, Carlo Enrico Confalonieri^2^, Ciro Esposito^2,3^

^1^Nephrology and Dialysis, Centre Hospitalier Le Mans, Le Mans, France

^2^Unit of Nephrology and Dialysis, ICS Maugeri s.p.a. SB, Pavia, Italy

^3^University of Pavia, Pavia, Italy

^‡^These Authors contributed equally to this manuscript

Massimo Torreggiani [maxtorreggiani@hotmail.com](mailto:maxtorreggiani@hotmail.com)

Marco Colucci [marco.colucci89@gmail.com](mailto:marco.colucci89@gmail.com)

Carlo Enrico Confalonieri [carlo.confa@hotmail.it](mailto:carlo.confa@hotmail.it)

Ciro Esposito [espositociro56@live.it](mailto:espositociro56@live.it)

All the Authors have no conflict of interest to disclose.

^*^Address correspondence to:

Massimo Torreggiani MD, PhD

Nephrology and Dialysis

Centre Hospitalier Le Mans

194 Avenue Rubillard,

72000 Le Mans, France

email: [maxtorreggiani@hotmail.com](mailto:maxtorreggiani@homail.com)

Keywords: Petrus Leo, de urinis, murder, uroscopy, Lorenzo The Magnificent, Renaissance, Italy, Middle Age.

**Historical background**

Urine has always been regarded as a useful diagnostic tool by physicians. In the ancient world and until the 19^th^ century urines were the only body fluid that could be non-invasively examined and whose characteristics were associated to health and disease. Hippocrates (V-IV century b.c.) hypothesized that urines were a filtrate of the four elementary humors of the body; for Galen (II century) urines represented a filtrate of the blood; Theophilus Protospatharius (VII-IX century) was the first to write an entire treaty on urine; the Persian physician Avicenna (X-XI century) described in detail the art of examining urines (uroscopy) (1-3).

In the Medieval Age, in Italy, the Salerno School of Medicine gave rise to a diffused medical culture between the 10^th^ and 13^th^ century. The great body of knowledge collected during these centuries was later published between the 15^th^ and 16^th^ century under the title “*Articella*” (4). Great importance was given to the examination of urines and several prominent doctors of the Salerno School originally wrote, or translated from ancient treatises, on this topic: Magister Maurus, Urso of Calabria and, later, Egide de Corbeil (5) tried to add something new or imitate the great masters of the past (6). The Salerno School promoted the rediscovery of Greek and Arabian medicine in an innovative and propulsive effort, mostly thanks to the translations by Constantinus Africanus (4).

Egide de Corbeil was an authority in the field and diffused his teachings beyond Salerno, establishing himself in Paris, thus becoming a cornerstone of medical studies around Europe until the 18^th^ century (4).

In the Renaissance Age, Italy was divided into several States, every one of which at war with the others in order to maintain the predominance of its territory. The most important States of that time were: the Reign of Naples ruled by the Aragonensis, the Maritime Republics of Genoa and Venice which ruled the sea with their fleets, the Vatican State ruled by the Pope, the Milan Duchy governed by the Sforza family and the Republic of Florence under the illuminated guidance of Lorenzo De’ Medici, also known as the Magnificent, between 1469 and 1492. Lorenzo was extremely loved by his citizens and consolidated his power granting prosperity to his city and its domains. The Magnificent had outstanding diplomatic abilities and Francesco Guicciardini defined him: “the needle of the Italian balance” (7). Indeed, after his death, on April 8, 1492, the fragile balance of Europe was disrupted by the thirst of power of the sovereigns of France, Spain and the Holy Roman Empire who invaded and conquered vast areas of Italy. At Lorenzo’s court gathered all the most prominent intellects of the Renaissance Age in a fervent cultural environment that had no equals in the previous or subsequent times. Lorenzo was a munificent patron for several artists such as Sandro Botticelli, Michelangelo Buonarroti, Leonardo da Vinci and for several literates, united in the Neoplatonic Circle, such as Angelo Poliziano, Marsilius Ficinus and Giovanni Pico della Mirandola, .

In the 15^th^ century Florence, studying and practicing medicine was a mean to move up on the social ladder and doctors who qualified for government office had the opportunity to find themselves in positions of political preminence (8). Most doctors took part in setting the tone of the Florentine cultural life (9) developing a New Galenic Academy devoted to criticizing the works of the Arabs (10).

Here, we will describe Petrus Leo’s life and interests, eventually discussing the events that led to his death.

**Petrus Leo: the formation years and its place at Lorenzo De’ Medici’s table.**

Petrus Leo (also known as Pierleone Leoni or Piero Leoni) was born around 1445 in Spoleto from a noble family (11). Not much is known about his childhood but it is believed that he studied medicine in Rome. For sure, while in Rome in 1475, he was offered a teaching position as *artium et medicine doctor* at the University of Pisa for three years (12, 13). Probably, coming from the Vatican State, he had some acquaintances and became popular among the highest grades of the clergy. The documents about Leo’s salary testify the importance of its role in the University and this position put him in contact with other prominent personalities such as Marsilius Ficinus and Lorenzo De’ Medici. At first, the relationship with the Magnificent was merely epistular as Lorenzo was the patron of *Studio Pisano* and had the last word in all the university affairs. In the years between 1478 and 1482 Petrus was again in Rome and probably served as one of the Pontifex’s doctors. In 1482 he returned to Pisa with an increased salary that reached 1000 florins starting from 1485. His fame allowed him to have some benefits to his contract, such as paid days off for businesses related to his medical activity (14), which allowed him to travel from time to time to Florence. It was at that time that he became Lorenzo De’ Medici personal doctor (13). From 1487 to 1490 Petrus was in Rome (15) and the University of Pisa tried in vain to call him back to teach in its *Studio* (14, 16). Petrus Leo never lost contact with the Magnificent who became a sort of protector for him. The following years were spent teaching in Padua until Petrus returned to Florence in 1491 were his fate was waiting for him on April 9^th^, 1492 (11, 17).

Lorenzo de’ Medici suffered from gout and Petrus tried to relieve his pain with his medical knowledge: this is attested by the fact that the eminent doctor, in January or February 1492, sent to Lorenzo a letter describing the beneficial properties of Tuscan thermal water (*Descrittione della proprietà et qualità del Bagno al Morbo*) (18, 19). Because Petrus Leo was the Magnificent’s personal physician, he was renown as first among Italian doctors according to Francesco Guicciardini (20).

However, Leo’s figure has been long debated. For instance, still at the beginning of the 20^th^ century, Thomas Mann, in his only theatrical piece “Fiorenza”, describes Leo as a man with white beard, quite eccentric, dressed like a charlatan or a wizard (21). This ungenerous description derives from Leo’s neoplatonic acquaintances (see below) and fits well with the rigorousness of a late 19^th^ century German author which could not stand interests other than science for medical doctors, particularly astrology. A vision probably derived from Paolo Giovio’s *Elogia* (22) which depicted Petrus as interested in kabbalah and astrology.

Nevertheless, we must look at Leo’s library to get an insight into his academic background and interests. It is believed that Leo’s library contained more than 190 texts (23) but this number seems an underestimation (24).

Among his books there were Latin as well as Greek, Arabian or Hebrew textbooks, some of them, translated by personalities surrounding the neoplatonic circle and copied by Petrus himself (13, 25). Petrus Leo was a strong advocate of Greek classics such as Galen and Hippocrates but, as a man of his time, he also indulged in astrology and kabbalah, supported by his friends Marsilius Ficinus and Giovanni Pico della Mirandola. It is important to note, however, that Petrus confined his magical interests to his private life and no mention of them was apparent during his teaching classes at the university or in his written production (26).

Besides a number of letters addressed to Lorenzo de’ Medici, we have two Leo’s works: one pharmacological treatise, *Opus medicarum curationum*, which mainly describes evacuative remedies and a transcription of the last year of lessons in Padua entitled *Recollectiones de febribus* or *De differentiis, causis et signis febrium* (27). Other manuscripts have been attributed to Petrus Leo who copied entire books or modified pre-existing texts adding notes (25, 28, 29). This is also testified by the fact that Pope Paul III encouraged one of Petrus’ nephews to print his uncle’s manuscripts judging them particularly useful (13). The only Leo’s printed work was the De urinis, a treatise about uroscopy written around 1478 and published in Venice in 1514 (see below).

**Between Earth and Heaven: the culture of Petrus Leo.**

During the years spent teaching in Pisa and practicing medicine at the court of the Magnificent, Petrus had the chance to be in contact with an incredibly fertile intellectual environment, soon becoming friend and cooperating with the most prominent personalities of the Florentine Renaissance. In particular, a close friendship with Marsilius Ficinus is testified by their correspondence, spanning over thirteen years, from 1478 to 1491 (30, 31). Petrus became distinguished member of the Neoplatonic Academy, being honored by Ficinus with the appellative “complatonicus” (32) and mentioned among the *familiares* of the Academy, along with Angelo Poliziano and Giovanni Pico della Mirandola (33). The spread of ideas in this circle had deep influence on Petrus's thought, bringing him in contact with ancient texts of classic authors such as Plotinus, Proclus Lycaeus, Pseudo-Dionysius the Aeropagite and, of course, Hippocrates and Galen (34). His precious contribution in revising Ficino's comment to Plotinus' Enneads and a lost Poliziano's translation of Hippocrates and Galen is acknowledged in various letters (35, 36) between the members of the Academy. Also, Petrus was primarily interested in rediscovering ancient medical texts: he estabilished a vast private librariy in Padua (37), later moved to Spoleto (13) after his death, which Poliziano described with admiration in a July 20^th^, 1491 letter to the Magnificent (29, 38). Manuscripts as *De dogmate Platonis et Hippocratis* and other treatisies “*de rebus medicinalibus*” by Galen (23, 28) played a prominent role in Leo's medical thought and his commitment in bringing to light the “true” Hellenic Galen. However, medical texts were just a part of his library, encompassing more than 200 manuscripts and 40 prints (23, 28). Petrus, described by Marsilius Ficinus as “v*ir cupidissimus secretorum*” [man interested in every secret of nature] (39), had a rich collection of astronomy, astrology, cabalistic and magic treatises written by Arabic and Jewish authors, such as the *Sefer Yesirà*, one of the first Hebrew mystic texts (23, 28). In the neoplatonic view of magic as “*pars practica scientiae naturalis*” advocated by Ficinus and Pico: Magic and astrology were considered a way to understand the intimate interpenetration between God, celestial bodies and material things; however, a sage could exploit this knowledge to escape the fate and exert free will (40). However, Petrus always maintained medicine and astrology clearly separated in his writings. Even under the heavy influence of the Florentine circle, Petrus was capable of developing an original interest for the work of philosophers like Nicholas of Cusa and Ramon Llull, almost completely neglected by Ficinus and Pico (41). Especially the doctrine of Raymond Llull, probably borrowed from Nicolas of Cusa (42), had an outstanding impact on Petrus’s thought and scientific approach. The A*rs Magna*, also known as a*rs combinatoria*, based upon the platonic view of a finite conceptual world, exploited the representation of highest concepts through single letters or signs, to combine them in every possible way, leading to the Truth (43). This methodology would become the cornerstone of the most fortunate original scientific writing of Petrus Leo, *De urinis*.

**Petrus Leo’s mysterious death**

In 1492 Lorenzo de’ Medici died. As already said, the Magnificent was affected by gout, as most of his family and descendants (44, 45) and in his 43^rd^ year of life the disease was so severe that nothing seemed to relieve him but thermal baths. For this reason, he used to frequent some thermal stations in Tuscany but in the spring of 1492 an ulcer due to gout got infected and caused gangrene. Thus he was taken to the Medicean mansion on the hill of Careggi in Florence where he died on April 8 (20). This is the traditional version of Lorenzo’s death, but a more recent study hypothesized that the Magnificent suffered from acromegaly and not gout (46).

Florence patron’s death was a shock for the entire Italian peninsula and for the continent as well because the delicate balance acquired during Lorenzo’s reign among the different European states was suddenly disrupted. Such a loss obviously elicited several different reactions, from grief to rage and suspect. According to a letter sent by Benedetto Dei to his uncle on April 14, 1492, Petrus was informed of Lorenzo’s severe health condition while in Milan and thus immediately left for Careggi (47). Upon his arrival on April 6, he discovered that the Magnificient had been treated with hot remedies while, instead, he needed cold treatments. He tried to his best to cure Lorenzo and he was able to prolong his survival for one day. When Petrus knew about Lorenzo’s death, rumors claimed that he lost his mental sanity and the following day he was found drowned in a well in San Gervasio, not far from Florence. This, apparently, happened because Petrus Leo had always sustained that gout could not kill the prominent Medicean and because all Leo’s certainties about his medical art suddenly failed (47). However, the suicide theory, sustained even by Poliziano (48), was later confuted. There was a suspicion of poisoning to explain Lorenzo’s death and so several Authors sustained that Petrus was killed as per Lorenzo’s son, Piero, order to find a scapegoat to calm the public opinion (13). Calcoldila (49) and Burchard (50) openly accused Piero while other Authors, such as Allegretti or Scipione Ammirato (48), used more caution, given the relevance of the de’ Medici family, asserting that Petrus was thrown in the well by two unknown Lorenzo’s relatives. Leo’s death notice spread to Italy and arrived in Rome on April 10 (50). The event had such a resonance that Guicciardini included Magister Leo’s death among the bad omens the preceded or followed the Magnificent’s passing (20). Scipione Ammirato, instead, finds in Leo’s death the inaugural act of Piero de’ Medici’s reign, mourning Leo as “excellent physician” (51).

**References**

1. Armstrong JA; Urinalysis in Western culture: a brief history. Kidney Int 2007; 71(5):384-7.

2. Kouba E, Wallen EM, Pruthi RS; Uroscopy by Hippocrates and Theophilus: prognosis versus diagnosis. J Urol 2007; 177(1):50-2.

3. Eknoyan G; Arabic medicine and nephrology. Am J Nephrol 1994; 14(4-6):270-8.

4. Pasca M; The Salerno School of Medicine. American Journal of Nephrology 1994; 14(4-6):478-482.

5. Oldoni M; Uroscopy in the Salerno School of Medicine. Am J Nephrol 1994; 14(4-6):483-7.

6. Angeletti LR, Gazzaniga V; Theophilus’ Auctoritas: The Role of De urinis in the Medical Curriculum of the 12th–13th Centuries. American Journal of Nephrology 1999; 19(2):165-171.

7. Elam C; Art and Diplomacy in Renaissance Florence. RSA Journal 1988; 136(5387):813-826.

8. Park K; Doctors in Florentine Society. Doctors and Medicine in Early Renaissance Florence. Princeton University Press: 1985, 151-187.

9. Park K; Doctors in Florentine Culture. Doctors and Medicine in Early Renaissance Florence. Princeton University Press: 1985, 188-236.

10. Park K; Conclusion. Doctors and Medicine in Early Renaissance Florence. Princeton University Press: 1985, 237-240.

11. Bacchelli F; Piero Leoni. Dizionario Biografico degli Italiani. Treccani, Roma: 2005.

12. Verde AF. Lo studio fiorentino : 1473-1503; ricerche e documenti. Vol. 2 Vol. 2*.* Istituto Nazionale di Studi sul Rinascimento, Firenze: 1973.

13. Guerra-Coppioli L; M° Pierleone da Spoleto, medico e filosofo. Note biografiche con documenti inediti. Bollettino della Regia Deputazione di Storia Patria per l'Umbria 1915; 21(2):387-431.

14. Pesenti T. Professori e promotori di medicina nello studio di Padova dal 1405 al 1509 : repertorio bio-bibliografico*.* Lint, Trieste: 1984.

15. Ficino M. Opera omnia [1576]*.* Bottega d'Erasmo, Torino: 1959.

16. Verde AF. Lo studio fiorentino : 1473-1503; ricerche e documenti. Vol. 2 Vol. 2*.* Istituto Nazionale di Studi sul Rinascimento, Firenze: 1973.

17. Rotzoll M; Pierleone da Spoleto: vita e opere di un medico del Rinascimento. Accademia La Colombaria. Serie studi. L.S. Olschki, Florence: 2000, 19.

18. Martínez Benavides MJ, Fernández Palomeque P, González Marrero JA; De urinis et pulsibus de Gilles de Corbeil y De urinis de Pierleone da Spoleto, un raro de 1514 conservado en la Biblioteca de la Universidad de La Laguna. Cuadernos de Filología Clásica. Estudios Latinos; Vol 26, N° 2 (2006) 2007.

19. Guerra-Coppioli L. Il Bagno a Morba nel Volterrano e m. Pierleone da Spoleto, medico di Lorenzo il Magnifico*.* Tipografia editrice S. Bernardino, Siena: 1915.

20. Guicciardini F, Palmarocchi R. Storie fiorentine dal 1378 al 1509*.* G. Laterza & Figli, Bari: 1931.

21. Mann T. Fiorenza*.* S. Fischer, Berlin: 1919.

22. Giovio P. Petrus Leonius*.* Michele Tramezzino: 1546.

23. Dorez L; Recherches sur la bibliothèque de Pier Leoni, médecin de Laurent de Médicis (2° article). Revue des Bibliothèques 1897; VII:81-106.

24. Radetti G; Un’aggiunta alla biblioteca di Pierleone Leoni da Spoleto. Rinascimento 1965(5):87-99.

25. Bacchelli F. Giovanni Pico e Pier Leone da Spoleto: tra filosofia dell'amore e tradizione cabalistica*.* L. S. Olschki: 2001.

26. Rotzoll M; Pierleone da Spoleto: vita e opere di un medico del Rinascimento. Accademia La Colombaria. Serie studi. L.S. Olschki, Florence: 2000, 48.

27. Pesenti T. Professori e promotori di medicina nello studio di Padova dal 1405 al 1509 : repertorio bio-bibliografico*.* Lint, Trieste: 1984.

28. Ruysschaert J; Nouvelles recherches au sujet de la bibliotèque de Pier Leone, médecin de Laurent le Magnifique. Bullettin de la Classe des lettres et des sciences morales et politiques de l'Académie riyale de Belgique 1960; Série 5 (4)(4):37-65.

29. Murano G; Tra scienza, astrologia e magia. Un nuovo manoscritto di Pierleone da Spoleto. Archivum mentis 2019(8):249-273.

30. Ficino M. The letters of Marsilio Ficino*.* Shepheard-Walwyn, London: 2015.

31. Ficino M. The letters of Marsilio Ficino*.* Shepheard-Walwyn, London: 2012.

32. Ficino M. Opera omnia [1576]*.* Bottega d'Erasmo, Torino: 1959.

33. Ficino M. Opera omnia [1576]*.* Bottega d'Erasmo, Torino: 1959.

34. Rotzoll M; Pierleone da Spoleto: vita e opere di un medico del Rinascimento. Accademia La Colombaria. Serie studi. L.S. Olschki, Florence: 2000, 25-43.

35. Poliziano A. Prose volgari inedite e poesie latine e greche edite e inedite*.* G. Barbèra, Firenze: 1867.

36. Ficino M. Opera omnia [1576]*.* Bottega d'Erasmo, Torino: 1959.

37. Branca V. Poliziano e l'umanesimo della parola*.* G. Einaudi, Torino: 1983.

38. Poliziano A. Prose volgari inedite e poesie latine e greche edite e inedite*.* G. Barbèra, Firenze: 1867.

39. Ficino M. Opera omnia [1576]*.* Bottega d'Erasmo, Torino: 1959.

40. Garin E. Lo zodiaco della vita : la polemica sull'astrologia dal Trecento al Cinquecento*.* Laterza, Roma; Bari: 2007.

41. Rotzoll M; Pierleone da Spoleto: vita e opere di un medico del Rinascimento. Accademia La Colombaria. Serie studi. L.S. Olschki, Florence: 2000, 37.

42. Rotzoll M. "Un certo vescovo da quelle parti ...": die Cusanus-Handschriften in der Bibliothek des Medici-Arztes Pierleone da Spoleto in Nicolaus Cusanus zwischen Deutschland und Italien: Beiträge eines deutsch-italienischen Symposiums in der Villa Vigoni*.* Akademie Verlag, Berlin: 2002.

43. Yates FA; The Art of Ramon Lull: An Approach to It through Lull's Theory of the Elements. Journal of the Warburg and Courtauld Institutes 1954; 17(1/2):115-173.

44. Fornaciari G, Giuffra V; [Rheumatic diseases at the court of the Medici of Florence: the so-called "gout" of the Medici]. Reumatismo 2009; 61(3):229-37.

45. Costa A, Weber G; [Pathological skeletal changes in Cosimo de' Medici (the elder), Piero the Gouty, Lorenzo the Magnificent and Julian, Duke of Nemours]. Archivio "de Vecchi" per l'anatomia patologica e la medicina clinica 1955; 23(1):1-69.

46. Lippi D, Charlier P, Romagnani P; Acromegaly in Lorenzo the Magnificent, father of the Renaissance. Lancet 2017; 389(10084):2104.

47. Frati L; La morte di Lorenzo de' Medici e il suicidio di Pier Leoni. Archivio Storico Italiano 1889; 4(173/174):255-260.

48. Roscoe W, Strahan A, Cadell T, Edwards J, Roscoe T. The life of Lorenzo de' Medici, called the Magnificent*.* Printed by A. Strahan, Printers Street, for T. Cadell, jun. and W. Davies, in the Strand and J. Edwards, Pall-Mall, London: 1800.

49. Bandini AM. Collectio veterum aliquot monimentorum ad historiam praecipue litterariam pertinentium*.* Michael Bellotti, Arezzo: 1752.

50. Burchard J. Johannis Burchardi Diarium sive rerum urbanarum commentarii (1483-1506). 3 vol. Leroux, Paris: 1885.

51. Ammirato S. Istorie fiorentine ridotte all' originale e annotate dal professore Luciano Scarabelli*.* Pomba, Torino: 1853.
